# 3D diffusion model within the collagen apatite porosity: An insight to the nanostructure of human trabecular bone

**DOI:** 10.1371/journal.pone.0189041

**Published:** 2017-12-08

**Authors:** Fabiano Bini, Andrada Pica, Andrea Marinozzi, Franco Marinozzi

**Affiliations:** 1 Department of Mechanical and Aerospace Engineering, “Sapienza” University of Rome, Rome, Italy; 2 Orthopedy and Traumatology Area, “Campus Bio-Medico” University, Rome, Italy; University of South Carolina, UNITED STATES

## Abstract

Bone tissue at nanoscale is a composite mainly made of apatite crystals, collagen molecules and water. This work is aimed to study the diffusion within bone nanostructure through Monte-Carlo simulations. To this purpose, an idealized geometric model of the apatite-collagen structure was developed. Gaussian probability distribution functions were employed to design the orientation of the apatite crystals with respect to the axes (length L, width W and thickness T) of a plate-like trabecula. We performed numerical simulations considering the influence of the mineral arrangement on the effective diffusion coefficient of water. To represent the hindrance of the impermeable apatite crystals on the water diffusion process, the effective diffusion coefficient was scaled with the tortuosity, the constrictivity and the porosity factors of the structure. The diffusion phenomenon was investigated in the three main directions of the single trabecula and the introduction of apatite preferential orientation allowed the creation of an anisotropic medium. Thus, different diffusivities values were observed along the axes of the single trabecula. We found good agreement with previous experimental results computed by means of a genetic algorithm.

## Introduction

Transport phenomena have a fundamental role in biological tissues, ensuring the supply of nutrients and the disposal of waste products. Diffusion of water is very sensitive to the local environment in tissues and is affected also by their packing geometry. The study of mass transfer could provide fundamental insights about the structure of biological tissues and to this aim, we investigated the water diffusion within the nanostructure of human femur head samples [1–2]. Bone is a highly specialized connective tissue which properties are influenced by its hierarchical structure. At nanoscale level, bone can be defined as a complex and ordered nanocrystal-reinforced composite. It consists of an organic matrix of mainly type I collagen fibrils [3], non-collagenous proteins and lipids, a crystal phase of calcium apatite and water [4]. Extensive studies [5–7] have been performed to understand the size, shape and composition of the mineral crystals, as well as their spatial distribution, orientation within the collagen fibrils and entropic elasticity [8].

Water occupies about 10–25% of the whole bone mass [9] and, as the third major component in bone, its role has been widely studied [10–12]. It is commonly accepted that bone presents four levels of porosity, which are nested hierarchically one inside another: collagen-apatite (10 nm), canalicular (100 nm), lacunar (up to 8 μm) vascular (50 μm), and the intertrabecular porosity (up to 1 mm) [13]. At the nano-level, water exists in the form of bound water in the collagen network and tightly bound water in the mineral phase. Other water molecules are present between the collagen triple helical molecules when mineral is absent or present in small amount [4]. The water in the collagen structure was classified into 5 regimes characterized by increasing water concentration. Regime V is characterized by free water between the fibrils and for the purposes of the present study, we considered the behaviour of free water within collagen-apatite network.

Marinozzi and his collaborators [14–16] carried out a study based on the swelling of a single dehydrated trabeculae from human femur head during water imbibition. The trabeculae were obtained from specimens of cancellous bone with moderate coxo-arthritis. Each specimen was extracted from a sample along the main stress trajectories in the loaded femur. Investigating the swelling over time along the three natural axes (Length L, Width W and Thickness T) of the plate—like trabecula, this allowed to achieve information about the water diffusion from external surfaces to the internal structure of the specimen.

Subsequently a 3D analytical model [14] of the water uptake was developed and it was used to predict the apparent diffusion coefficients along the three axes of plate-like trabeculae (Length L, Width W and Thickness T) by means of a genetic algorithm. A great difference was found among the three diffusion coefficients i.e., [D_L_ = 1.03·10^−9^, D_W_ = 1.26·10^−10^, D_T_ = 1.16·10^−11^ (m^2^·s^-1^)]. The major diffusivity was in the longitudinal direction, while minor values, with one and two orders of magnitude than D_L_, corresponded to D_W_ and respectively D_T_.

This study is intended to achieve information about the fluid transport dynamics within the collagen-apatite porosity. The main scope is to develop a 3D geometric model of the nanostructure by means a system of equations that describes the diffusion phenomenon at this level of porosity. The work aims to create a bridge between the diffusion studies at the molecular level [17] and the lacunar—canalicular scale [13].

Here, we considered continuum mechanics theory concepts, utilizing a staggered model [18–19] of the mineralized collagen fibril. Hence, we calculated the effective diffusion coefficient within bone nanostructure model considering the steric hindrance of the biological components [20–21]. The results were then compared with the available data obtained from the genetic algorithm [14].

## Materials and methods

### Model geometry

**Fig 1:**
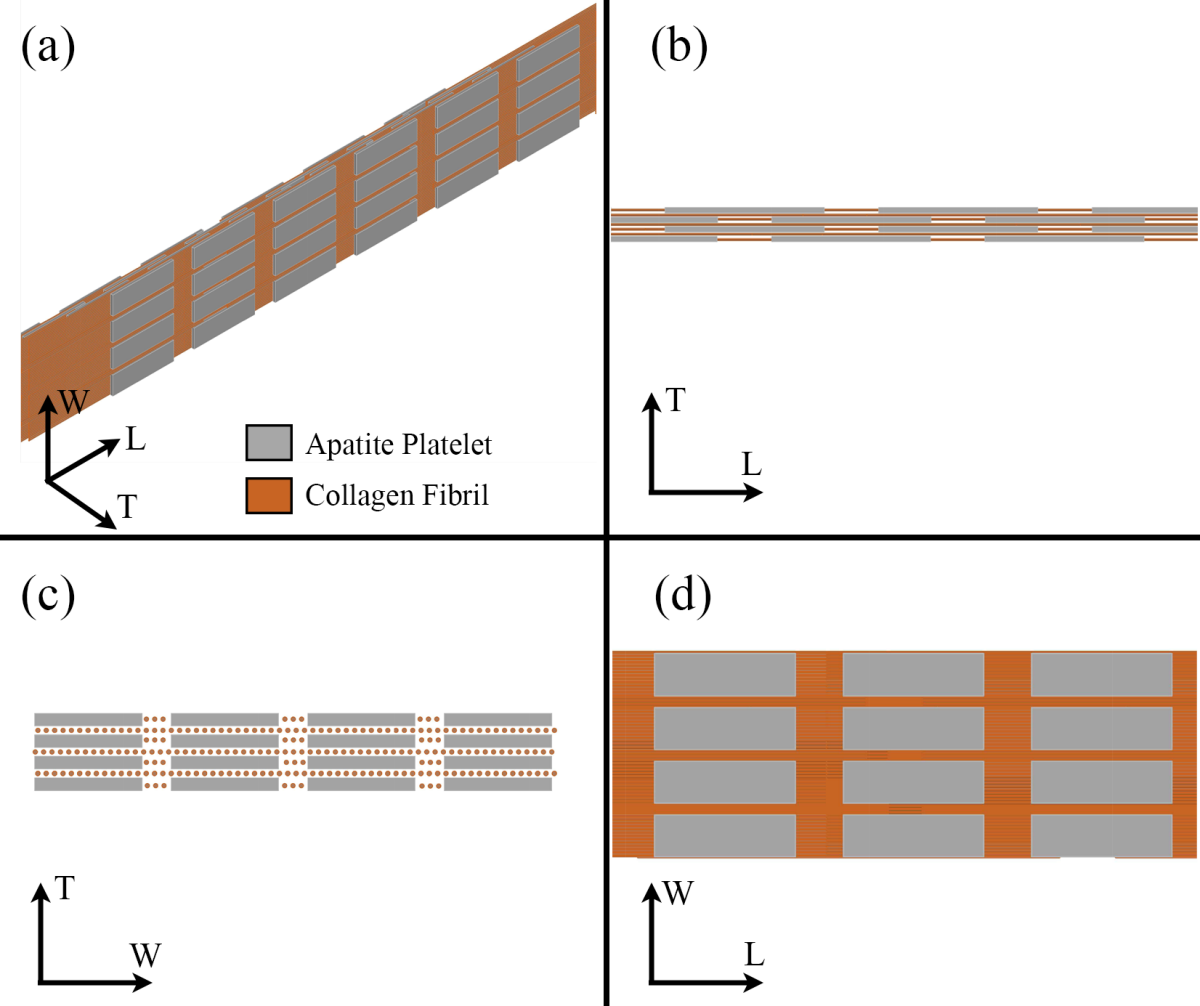
Model of the mineralized collagen fibril: (a) 3D view of apatite platelets embedded in a collagen matrix, (b) longitudinal plane, (c) cross section and (d) frontal plane.

**Fig 2:**
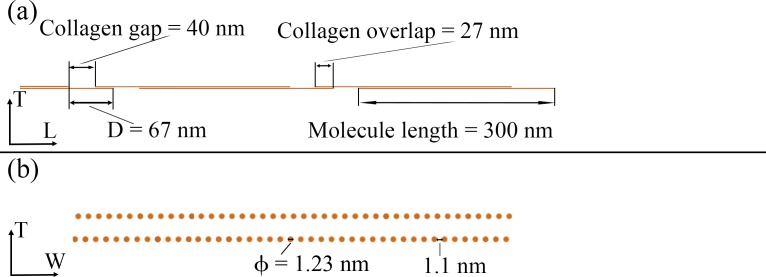
Schematic representation of collagen molecules staggered arrangement based on Hodge et al. [18]: (a) longitudinal plane and (b) cross section of the nanostructure.

**Fig 3:**
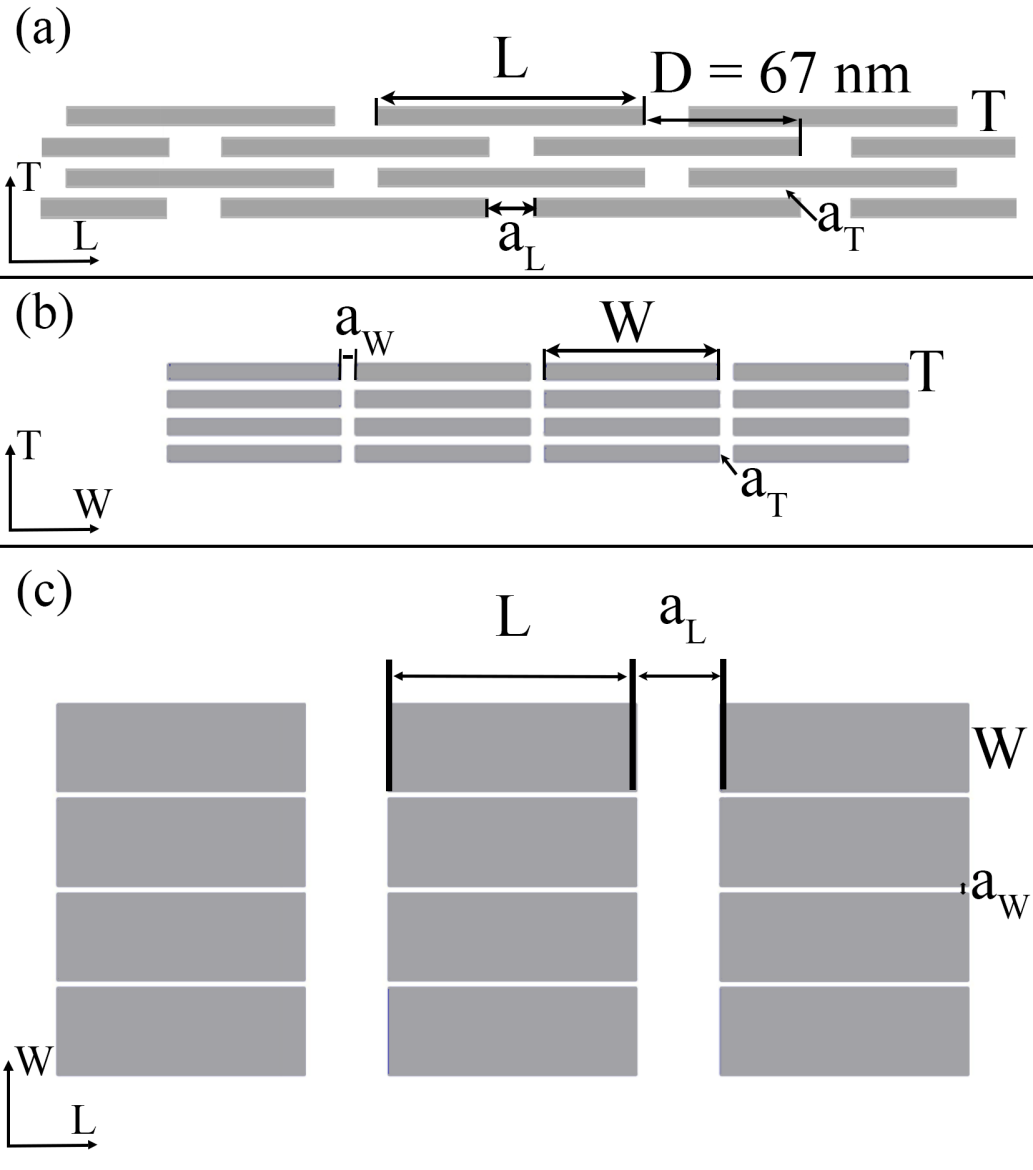
Sketch not drawn to scale of the apatite array considering a staggered arrangement in the axial direction (a) and a distribution of parallel layers in the transverse direction of the fibrils (b, c). In (a) the longitudinal plane of the apatite matrix is shown, indicated as LT plane. In (b) is represented WT plane, while in (c) the frontal plane is illustrated (LW plane). The principal geometric variables are also highlighted.

A 3-D model of the embedded apatite crystals within the collagen fibrils has been developed in CAD environment (Autodesk AutoCAD 2016, San Rafael, CA) according to the model proposed by Hodge et al. [18] and Jäger et al. [19]. The elementary components of the mineralized collagen fibril are characterized as follows (Fig 1):

1. Collagen: Type I collagen molecules self-assemble into triple helical tropocollagen molecules, which are approximated by cylinders with 300 nm length and 1.23 nm diameter [4–5]. Adjacent collagen molecules are staggered in the axial direction of the fibril by a periodic distance, D = 67 nm, generating a characteristic pattern of gap zones 40 nm long and overlap zones 27 nm long within the fibril. In a cross section of the fibril (Fig 2), tropocollagen molecules are arranged in a quasi-hexagonal array [22].
2. Apatite mineral: we assume that all apatite crystals are plate-like shaped, distributed in a staggered arrangement in the longitudinal direction of the collagen fibril and in parallel layers in the transverse directions of the fibril (Fig 3).

In the present work, we considered two different degrees of mineralization (i.e., V_f_A_ = 0.25 and V_f_A_ = 0.43). For each model, which is characterized by a constant apatite volume fraction, we hypothesized the length (L) and width (W) of the platelets spanning across the extensive range of values indicated in literature (Table 1). Conversely, the mineral thickness (T) is maintained constant at 3.5 nm [19]. In the width and thickness directions, distances between neighbouring apatite platelets (a_W_ and a_T_ respectively) are of the same order as crystal thickness [23]. In the longitudinal direction, the distance between two consecutive crystals, i.e. a_L_, is set in order to satisfy the following constraint [19]:

$$\left(\frac{L+a_{L}}{2}\right)=D$$ (1)

where D is the length of the axial collagen period.

**Table 1:** Range of values for the apatite platelets size.

Parameter	V_f_A_ = 0.25	V_f_A_ = 0.43	References
**L**	(70–110) nm	(90–130) nm	[37]
**W**	(9–60) nm	(9–60) nm	[37]
**T**	3.5 nm	3.5 nm	[19]
**a**_**L**_	(24–64) nm	(4–44) nm	[19]
**a**_**W**_	(8–12) nm	(4–12) nm	[22]
**a**_**T**_	(2–3.5) nm	(1.5–3.5) nm	[22]

We assumed that the values of the crystal length (L) and the distances between platelets (i.e., a_W_ and a_T_) are characterized by Gaussian Probability Density Function (PDF). For each variable, we extracted 100 samples from the corresponding PDF's and used them subsequently as input parameters in the Monte Carlo Method. For each extracted sample of the above geometric parameters, the width (W) of the platelet was determined in order to maintain constant the apatite volume fraction (V_f_A_) for each model, calculated as follows:

$$V_{f_{A}}=\frac{LWT}{\left(L+a_{L}\right)\cdot \left(W+a_{W}\right)\cdot \left(T+a_{T}\right)}$$ (2)

### Unit cell inclination

In this paper, we identified as unit cell the mineralized collagen fibril that is bone’s building block at an ultrastructural level. For this purpose, we introduced a local coordinate system aligned with the platelet and a global coordinate system aligned with the axes of the single trabecula.

Georgiadis et al. [24] presented a first attempt to investigate by means of 3D scanning small-angle X-ray scattering (sSAXS) the spatial organization of the ultrastructure in human trabecular bone, in the context of the underlying trabecular microstructure.

The 3D orientation maps indicate that trabecular bone ultrastructure in healthy human vertebral bone is organized in different orientation domains, several tens of micrometers in size, which is in line with conclusions based on SAXS data that can be drawn from 2D orientation maps from trabecular structure in the femoral head [25].

Tomography shows that the crystals in tendon collagen are aligned in a series of platelets whose crystallographic *c-axes* are nearly parallel to one another and to the long axes of the collagen molecules and fibrils with which they associate [26]. Variations in this orientation of individual crystals range ± 20 degrees. The generally parallel alignment of the crystallographic *c-axis* of the mineral platelets and the collagen long axes is consistent with results from numerous other study [23].

Since the crystals are a positional marker for the spatial location of the hole and overlap zones of collagen, the finding of coincident, parallel crystals in neighbouring fibrils provides clear evidence that different fibrils may be strictly organized, themselves, as collagen aggregates, assembles, and becomes stabilized by cross-linking throughout the osteoid [27]. This conclusion for bone collagen supports the suggestion that many fibrils together may be aligned in a coherent manner as reported in an earlier study by electron diffraction of calcifying avian tendon [28].

The parallel nature of the platelet-shaped crystals, dictated by their specific association with collagen at the molecular level, is maintained at higher orders of structure and is mirrored in the fibril interactions [29].

In this study, the following spatial assumptions were adopted: (a) the three staggered platelets and the adjacent collagen composing the functional unit cell maintain their reciprocal parallelism independently of the cell inclination with respect to global coordinate system; and (b) at the nanoscale, adjacent elementary cells have a high degree of alignment with respect to each other, while a low degree of alignment is supposed at a larger scale (e.g. micrometer scale) [30].

**Fig 4:**
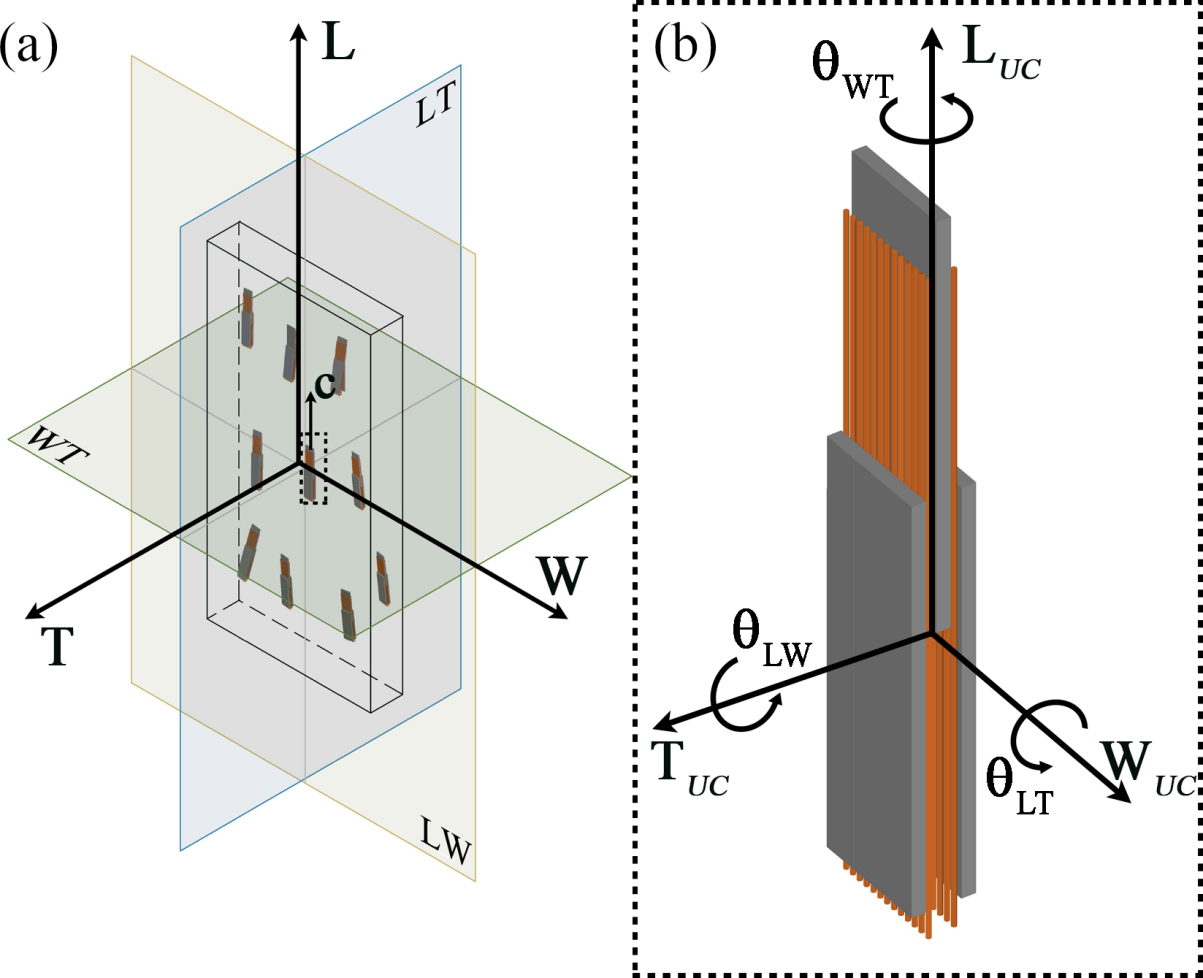
(a) Sketch not drawn to scale of the representative unit cell within a single trabecula. By hypothesis, the three platelets composing the unit cell (UC) maintain their reciprocal parallelism independently of the cell inclination with respect to global coordinate system. The *c-axis* of the apatite platelets is aligned with the collagen long axis and points into the longitudinal direction of the single trabecula [23]. The global coordinate system and the three orthogonal planes considered are illustrated. (b) The dashed rectangle represents a zoom of the UC composed of three apatite platelets embedded in the collagen matrix. The local coordinate system is shown.

The inclination of mineral with respect to the longitudinal axis L is identified by the angle θ_LW_ in the LW plane and θ_LT_ in the LT plane. In the WT plane, the inclination, specified by θ_WT_, is considered with respect to the W axis (Fig 4). Uncorrelated determinations of the three variables (θ_LW_, θ_LT_, θ_WT_) are obtained by random extraction, from a Gaussian distribution.

The preferential orientation of platelets is described by Gaussian PDF with mean μ and standard deviation σ. We assumed that, on average, the unit cell is aligned to the global coordinate system, and thus μ = 0 degrees. For the standard deviation we considered random values from a Gaussian distribution in the range from 0 degrees to 20 degrees [23]. In order to obtain a finite angle domain, numerical simulations were carried out with truncated Gaussian distributions in the interval [-2σ; +2σ].

### Diffusion coefficient

To interpret and compare the previous results [14], it was necessary to implement a model of the diffusion coefficient along the main axes of the single trabecula. Effective diffusion coefficient (D_eff_) is significantly lower than free water diffusion coefficient (D_0_), because of the collagen—apatite hindering geometry and porosity. For the self diffusion coefficient of water molecules we adopted the value computed by means of molecular dynamics within confined geometries such as nanopores, i.e. D_0_ = 2.66·10^−9^ m^2^·s^-1^ at 27°C [31].

We considered the following relation between D_eff_ and D_0_ [20–21]:

$$D_{eff}=D_{0}\cdot \frac{\delta \cdot \varphi }{\tau }$$ (3)

where φ is the porosity of the medium, δ is the constrictivity factor and τ is the tortuosity factor.

Constrictivity δ ϵ [0, 1] is a dimensionless parameter that represents the hindrance ensuing from the non-uniform width of the passageway between the hexagonal packing of collagen fibrils [20] and results influenced by the inclination θ. Therefore, it is estimated [32] by Eq (4):

$$\delta =\frac{\sqrt{\max\;cross\;\sec tion\;\cdot \;\min\;cross\;\sec tion\;}}{mean\;cross\;\sec tion}$$ (4)

The porosity is considered independent from the inclination of the elementary cell. Established a representative elementary volume, (***L*** + ***a***_***L***_)⋅(***W*** + ***a***_***W***_)⋅(***T*** + ***a***_***T***_), the porosity is defined as [22]:

$$\varphi =1-V_{f}$$ (5)

where V_f_ is the volume fraction of the unit cell and is expressed by Eq (6):

$$V_{f}=V_{f_{A}}+V_{f_{collagen}}=\frac{LWT}{\left(L+a_{L}\right)\cdot \left(W+a_{W}\right)\cdot \left(T+a_{T}\right)}+\frac{n_{c}\pi \cdot {\left(0.5d\right)}^{2}\cdot \left(L+a_{L}-0.6D\right)}{\left(L+a_{L}\right)\cdot \left(W+a_{W}\right)\cdot \left(T+a_{T}\right)}$$ (6)

where n_c_ is equal to the number of collagen fibrils, d is the diameter of the collagen fibrils and D is the axial period of the collagen fibrils, respectively.

The tortuosity (τ ≥ 1) is a dimensionless factor that accounts for the reduction of diffusive flux caused by the tortuous paths of the solute molecule, compared to the straight paths in an unrestricted aqueous medium [33]. Tortuosity is defined as follows [21, 34–36]:

$$\tau ={\left(\frac{l_{i}}{l}\right)}^{2}$$ (7)

where *l*_*i*_ is the effective path length and *l* is the Euclidean distance in the direction of flow between the path extremes.

The definition of tortuosity is adapted to the collagen-apatite network model. We assume that a concentration gradient exists along one of the three axes of the coordinate system at a time and the pore space allows the crossing of a single molecule of water at a time.

For simplification purposes, it is first analysed the tortuosity of flow paths in a medium with apatite platelets surrounded by a homogeneous matrix representing the collagen array. The tortuosity of streamlines in the collagen matrix is investigated subsequently without considering the presence of the mineral. The overall value of tortuosity (τ), introduced in Eq (3), is obtained combining both tortuosities, as indicated in Eq (8):

$$\tau =\tau_{Apatite}\cdot \tau_{Collagen}$$ (8)

where τ_Apatite_ is the tortuosity calculated in the mineral matrix and τ_Collagen_ is the tortuosity calculated in the collagen matrix.

### Determination of tortuosity

**Fig 5:**
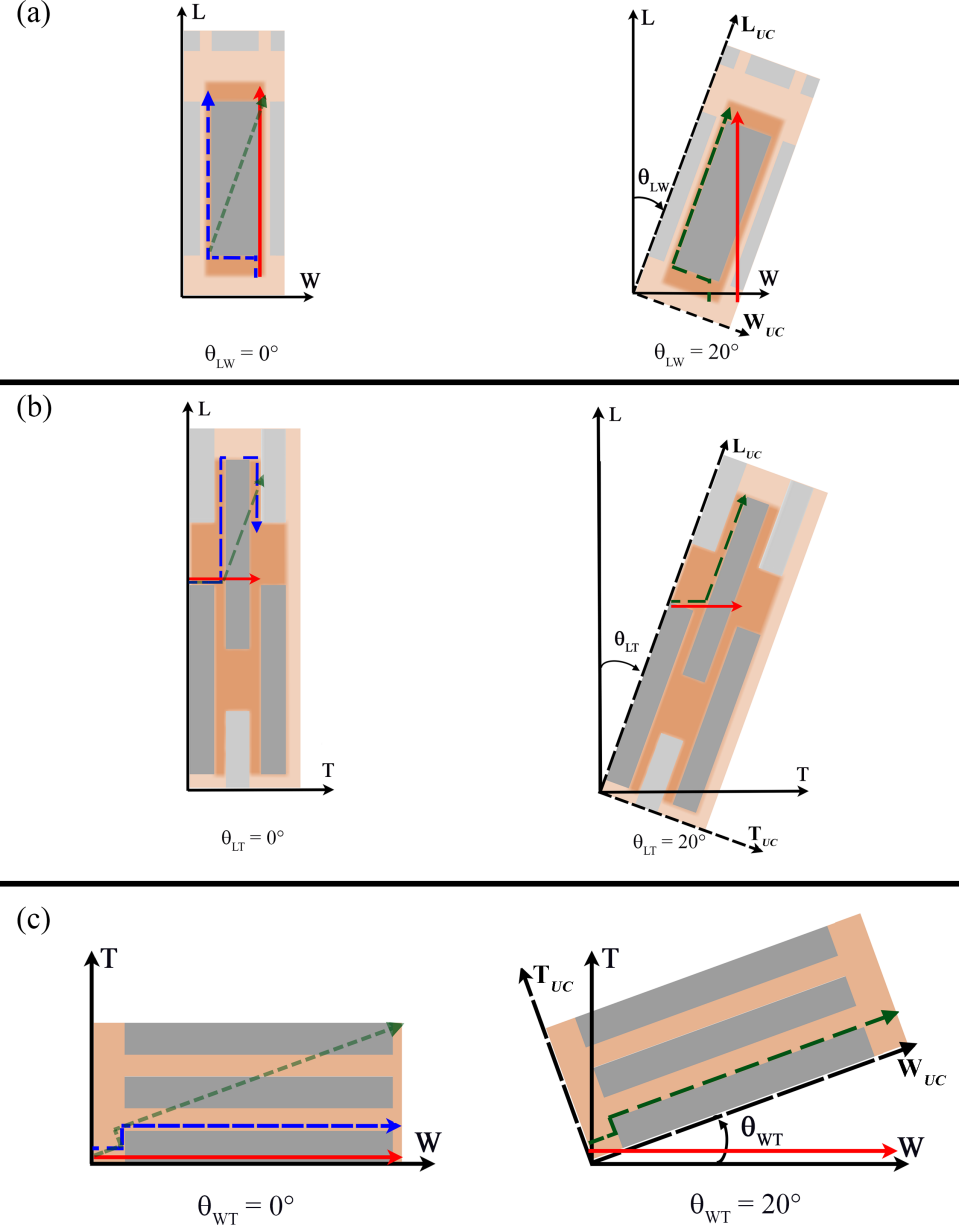
Two examples of aligned (θ = 0 degrees) and inclined (θ = 20 degrees) apatite platelets configurations are depicted in a) LW plane, b) LT plane and c) WT plane. The blue dashed line (at θ = 0 degrees) and the green dashed line (at θ = 20 degrees) represent the streamlines of water molecule within the apatite network along the investigated direction in order to highlight the primary influence of the tortuosity on the diffusion coefficient. Whilst the red continuous line indicates the Euclidean distance between the path extremes.

For each inclination of the functional unit cell, the tortuosity is carried out from Eq (7). With regard to the tortuosity relative to the mineral matrix, we analysed some representative streamlines within one unit cell of the apatite crystals in all of the three main planes of the coordinate system (i.e., LW, LT, WT). The effective path length is defined considering that in the homogeneous matrix the streamlines (Fig 5 blue and green dashed line), parallel to the diffusion gradient, represent the flow of the water molecule within the platelets network along the investigated direction. We calculated the path length by means of geometrical considerations taking into account the apatite size and inclination (θ_LT_, θ_WT_, θ_LW_). The Euclidean distance is defined as the straight line (Fig 5 red continuous line), parallel to the direction of the fluid path that connects the path extremes in absence of any obstacles represented by apatite minerals. For each configuration of the unit cell, multiples streamlines could be considered in order to reach the extremities of the unit cell. Therefore, we compute the average of all values of tortuosity relative to a specific orientation, as follows:

$$\tau_{Apatite}=\frac{1}{N}\sum_{i}\tau_{{}_{i}}$$ (9)

where *N* is the total number of flow paths relative to the specific orientation of the unit cell and *τ*_*i*_ is the tortuosity for the *i*^*th*^ streamline [34]. The value obtained from Eq (9) is then used in Eq (8).

The tortuosity within the collagen matrix is calculated through Eq (7) and considers that the cross-sections of the fibrils with respect to the axes of coordinate systems are elliptical as the collagen follows the mineral inclination. Therefore, the water molecule, given the densely packed structure of collagen, performs elliptical trajectories whose length (*l*_*i*_) is equal to the semi-perimeter of the ellipse, while the Euclidean distance (*l*) corresponds to the major axis of the ellipse.

### Determination of the diffusion coefficients

For each plane (i.e., LW, LT, WT), we investigated the diffusion along the two main orthogonal directions that characterize the plane. Monte Carlo Method (Mathematica 10, Wolfram, Oxfordshire, UK) was used in order to simulate the inclinations of the unit cell. The simulations were characterized by random extraction of values θ from a truncated Gaussian distribution, in the range from -2σ to +2σ.

The methods performed in order to compute the diffusion coefficient along one of the axes of the trabecula can be summarized as follows:

1. random extraction of the input parameters, i.e., apatite inclination tern (θ_LW,_ θ_LT_, θ_WT_) and mineral crystal size and distances between platelets (L, a_L_, a_T_ and a_W_) from the Gaussian PDF. Calculation of the mineral width W in order to satisfy Eq (2) and calculation of the longitudinal distance between platelets, a_L_, in order to satisfy Eq (1);
2. calculation of the tortuosity within the apatite matrix in function of the parameters previously set, following the procedure illustrated in the subsection “Determination of tortuosity”;
3. calculation of the constrictivity and tortuosity within the collagen matrix, applying Eq (4) and Eq (7) respectively in function of (θ_LW_, θ_LT_, θ_WT_);
4. calculation of the apparent diffusion coefficient by means of Eq (3);
5. repeating all previous steps until completing N realizations, where N = 100 is the imposed number of samples for the apatite platelets size and inclination respectively;
6. repeating all the previous step up to the imposed number of Gaussian PDF considered for the apatite inclination. In this study, we considered 100 different Gaussian PDF’s.

The output of the Monte Carlo simulations provides the diffusion coefficient for different configuration of mineral platelets along the orthogonal directions of the main planes of coordinate system (i.e. LW, LT, and WT plane). In this manner, information about the diffusivity along a specific direction is obtained from two planes. Therefore, we have been performed an average between the values of the diffusion coefficient resulting from the two different planes and competing to a specific apatite platelet configuration. Subsequently, we carried out the mean of means of the effective diffusion coefficients obtained for each Gaussian PDF considered for the mineral inclinations.

### Statistical analysis

We performed a nonlinear fit on the mean of means corresponding to each diffusion gradient in function of the standard deviations of the Gaussian PDF that describes the inclination of the apatite platelets in three main planes. For each value, it was considered also a confidence interval of 95 percent, computed multiplying the coverage factor *k* by the standard error (SE), calculated as follows

$$SE=\frac{\sigma }{\sqrt{N}}$$ (10)

where σ is the standard deviation and N is the number of observations.

A χ^2^ test was applied to the final values of the diffusion coefficients D_L_, D_T_, D_W_ in order to assess whether data is normally distributed. The three effective diffusion coefficients resulted normally distributed.

## Results

**Fig 6:**
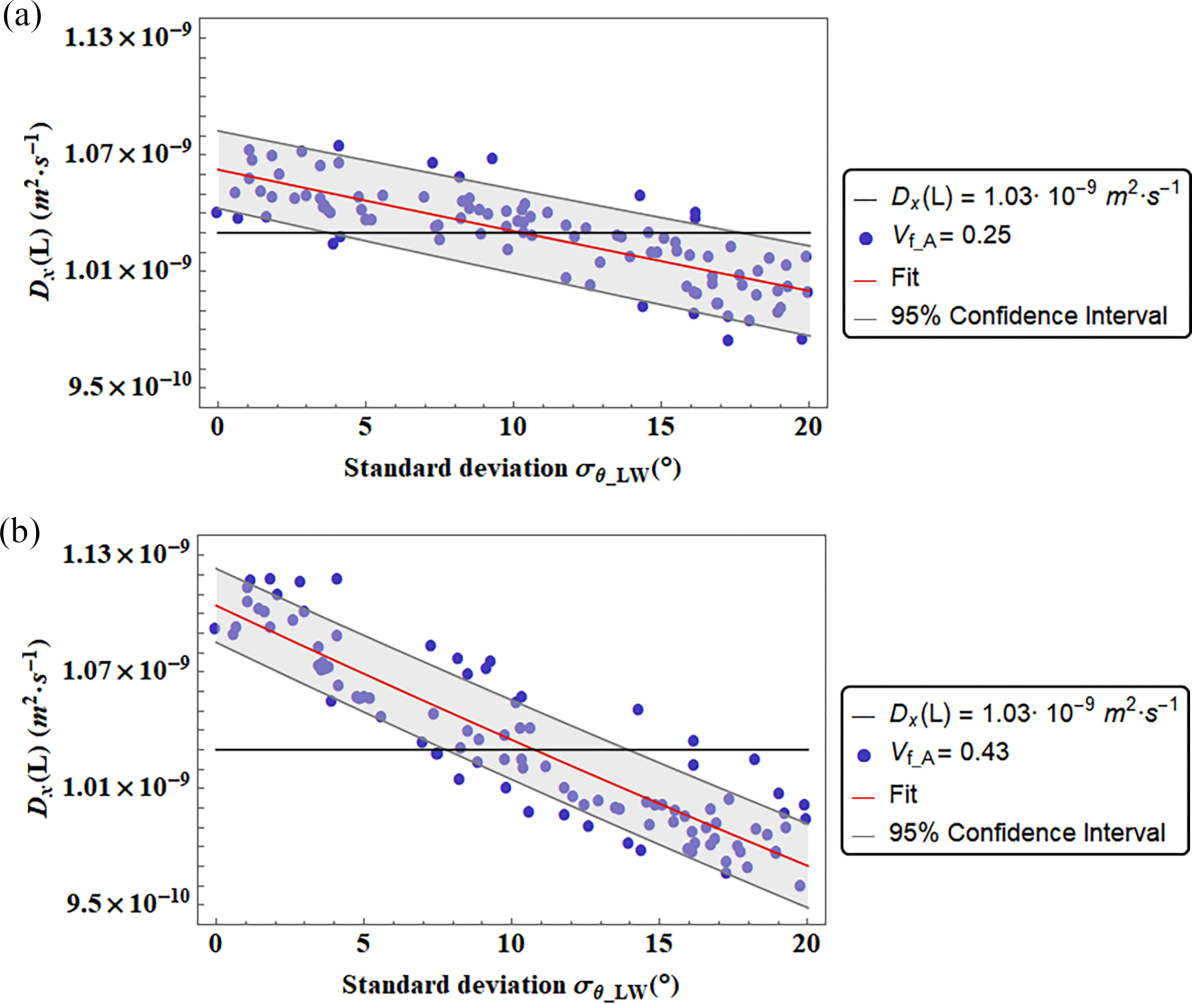
Diffusion coefficients D_L_ versus the standard deviation (σ_θ_LW_) of the Gaussian PDF that characterizes the apatite platelets inclination in the LW plane. The color bands represent the Confidence Interval at 95 percent. We reported also two different degrees of mineralization (V_f_A_ = 0.25, and V_f_A_ = 0.43). The continuous black line represent the Diffusion coefficient computed by means of a genetic algorithm [14].

**Fig 7:**
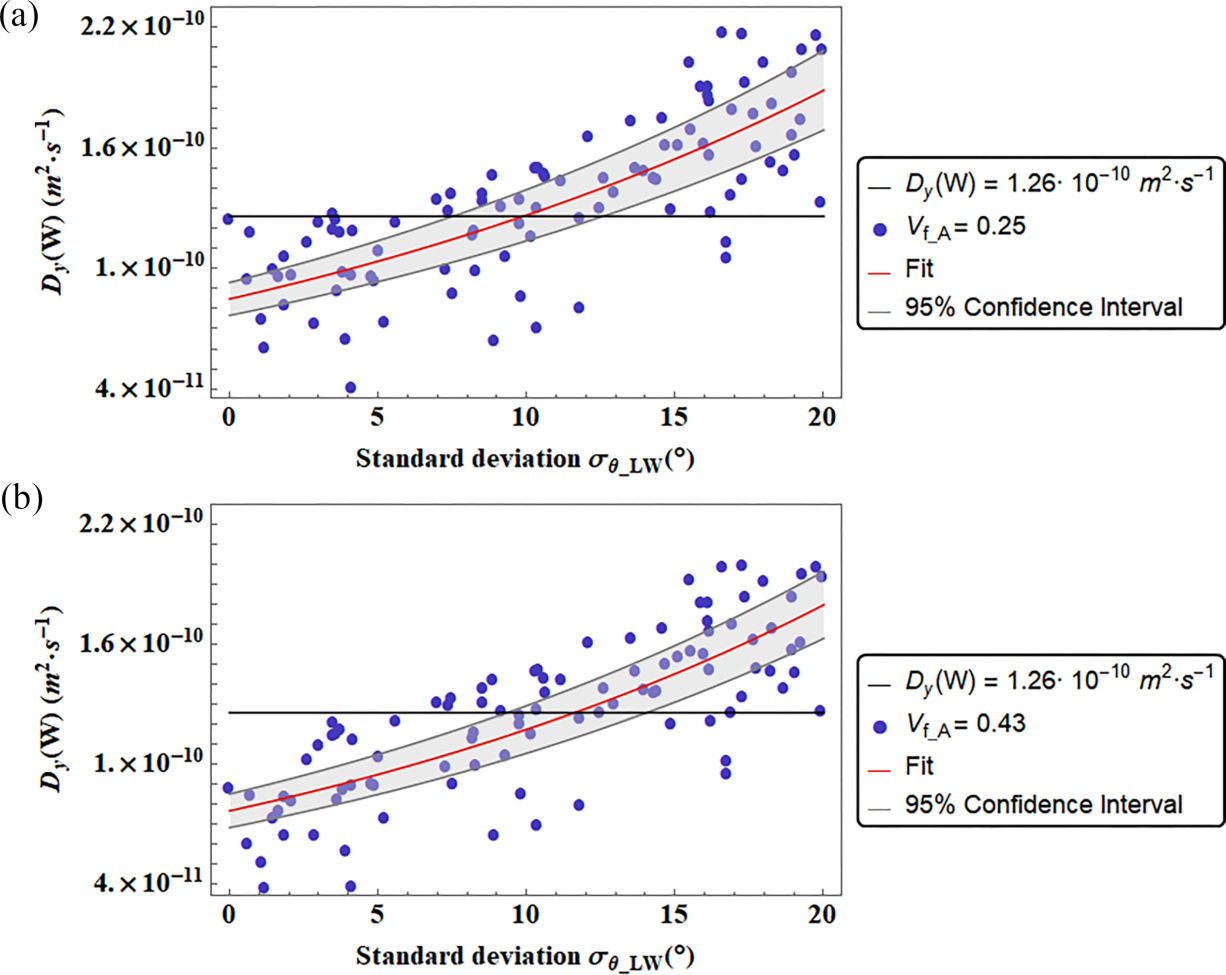
Diffusion coefficients D_W_ versus the standard deviation (σ_θ_LW_) of the Gaussian PDF that characterizes the apatite platelets inclination in the LW plane. The color bands represent the Confidence Interval at 95 percent. We reported also two different degrees of mineralization (V_f_A_ = 0.25, and V_f_A_ = 0.43). The continuous black line represent the Diffusion coefficient computed by means of a genetic algorithm [14].

**Fig 8:**
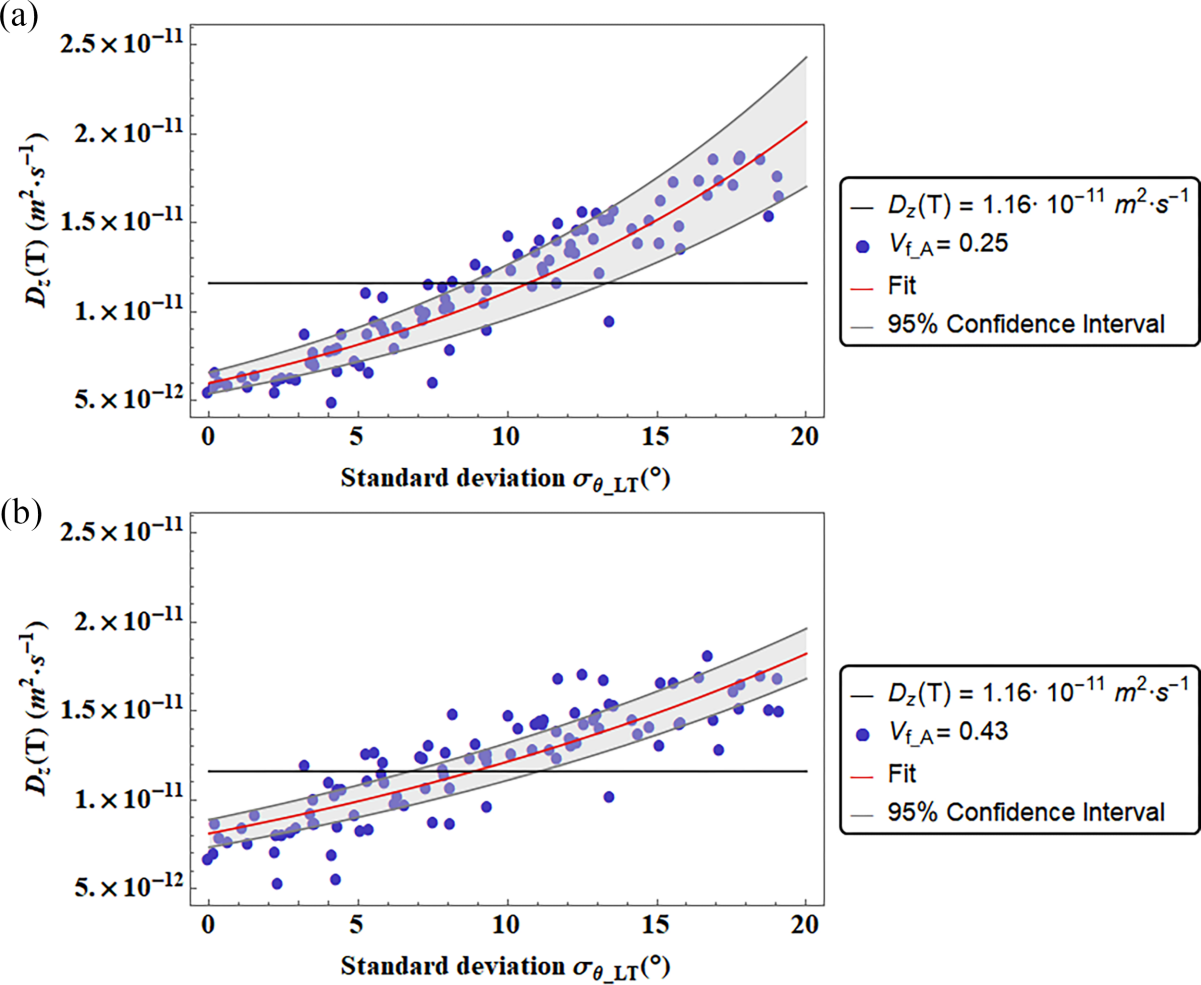
Diffusion coefficients D_T_ versus the standard deviation (σ_θ_LT_) of the Gaussian PDF that characterizes the apatite platelets inclination in the LT plane. The color bands represent the Confidence Interval at 95 percent. We reported also two different degrees of mineralization (V_f_A_ = 0.25, and V_f_A_ = 0.43). The continuous black line represent the Diffusion coefficient computed by means of a genetic algorithm [14].

In Figs 6 and 7 the diffusion coefficients are represented in function of the standard deviation of the Gaussian PDF that characterize the apatite inclination with respect to one of the main axes of the coordinate system. We illustrate the diffusivities D_L_ and D_w_ in function of the standard deviation σ_LW_ related to the Gaussian PDF of the mineral inclination θ_LW_, while the coefficient D_T_ (Fig 8) is represented in function of the standard deviation σ_LT_ related to the platelets inclination in the LT plane. We represented the diffusion coefficients in function of the standard deviation of the inclination from which it was observed a preeminent dependence.

Other representations of the diffusion coefficients are available in Supplementary material, i.e. D_L_ in function of the standard deviation σ_LT_ of the Gaussian PDF of the mineral inclination θ_LT_ in the LT plane (S1 Fig), D_W_ and D_T_ in function of the standard deviation σ_WT_ related to the apatite inclination in the WT plane (S2 Fig and S3 Fig).

The comparison between the coefficients obtained through Monte-Carlo methods and the genetic algorithm (Table 2) allows to identify the optimal arrangement of the mineral—collagen model that better mimics the experimental results [14]. Furthermore, it contributes to define the anisotropy of bone nanostructure.

**Table 2:** Standard deviation of the Gaussian PDF representing the inclination of the apatite platelet matching the diffusion coefficient in agreement to [14].

Diffusion coefficient	V_f_A_ = 0.25			V_f_A_ = 0.43		
	σ_LW_	σ_LT_	σ_WT_	σ_LW_	σ_LT_	σ_WT_
**D**_**L**_	10.3°	8.3°	10.5°	10.5°	9.8°	11.6°
**D**_**W**_	9.7°	6.5°	9.3°	11.6°	10°	11°
**D**_**T**_	10.8°	10.5°	11.8°	10.2°	8.8°	9.2°

## Discussions

The 3D geometric model within the bone nanostructure model provided significant results in light of the studies about the arrangement of apatite crystals in the collagen matrix [23, 37].

Although electron microscopic tomography and image reconstruction have been utilized to study the 3D spatial arrangement of mineral crystals within the fibril [23, 37], the resolution of this technique (4–6 nm) is well above what is needed to resolve the thickness of mineral platelets embedded in the densely packed collagen fibrils. Therefore, scarcely is known about the packing of mineral crystals in the radial direction of the collagen fibril. The models developed by the Authors of TEM and AFM studies [38] assume that the mineral crystals are randomly distributed in the cross section of fibrils, which is inconsistent with the numerical simulation presented here, the effective diffusion coefficient study [14], the AFM investigation [37].

Burger et al. [37] have suggested that a random distribution of the platelets within the fibril cross section must not allow platelet overlap and, thus, it is feasible only if the degree of mineralization is very low. In this study, Monte Carlo simulations highlight that, for a low degree of mineralization (V_f_A_ = 0.25), the model still presents a preferred orientation. It could be deduced that also for extremely low mineralization, a preferential orientation of apatite platelets is maintained.

The literature provides more information about the mineral arrangement in the longitudinal section of trabecula. Small-angle scattering tensor tomography study [39, 40] evidenced that, at the nanoscale, collagen fibrils, have a high degree of alignment parallel to the longitudinal axis of trabecula, which corresponds to the main stress direction. Different studies [4, 5, 23, 40, 41, 42] reported that the mineral *c-axis* is parallel to the longitudinal axis of collagen. Therefore, assuming an alignment between collagen fibrils and apatite crystal [39], the latter will have a preferential orientation, parallel to the main axes of trabecula. In the trabecular bone of the proximal human femur the apatite crystals need to be oriented such that they can resist the load from various directions. In Hong at al. [38] a random orientation of the platelets with respect to the whole trabecular bone coordinate system is suggested in order to resist the local stress state. In this study and in Marinozzi et al. [14–16], the preferential orientation of the mineral is considered with respect to the axes of a single trabecula and not with respect of the bone axes. Therefore, the random arrangement indicated by Tong et al. [7] and Hassenkam et al. [43] is referred to a larger scale (e.g. the whole trabecular bone). The single trabecula could be considered as a small region of local order indicating a stacking direction of the mineral plates.

A random arrangement of the apatite crystals is not consistent with Marinozzi et al. [14], Jäger et al. [19] and Burger et al. [37]. For further verification of this circumstance, we initially performed some simulations with the proposed model assuming that the orientation of the unit cell could have equiprobable orientations in the interval [-90°;+90°] with respect to the global coordinate system. Under this assumption, the diffusion coefficients computed along the three main axes of the trabecula resulted of the same order of magnitude, in neat contrast to the genetic algorithm results [14].

Therefore, in the present study, preferential orientation of the collagen-mineral matrix was considered with respect to the three main axes of the trabecula and this allows to match the results obtained with the genetic algorithm [14]. Oriented apatite configuration in the longitudinal direction finds agreement with Weiner et al. [4], Jäger et al. [19], Landis et al. [23], Liebi et al. [39] and Jaschouz et al. [40], but not with Tong et al. [7]. High resolution TEM [38] was performed to analyse the orientation of apatite crystals in murine femoral bone. The authors found that the mineral apatite was arranged with no preferential orientation. This discrepancy with our study could be due to the different species investigated (human *vs* murine), but also to a different scale of observation of the orientation randomness (local collagen fibril *vs* whole bone). The latter justification is valid also for Tong et al. [7].

The effective diffusion coefficient can be used to highlight the anisotropic structure of the collagen-apatite matrix, since it was calculated considering the influence of the geometric structure.

In the present work, quantitative information about the orientation of the mineral inclination with respect to the trabecula axes are provided for healthy human bone trabecular tissue, with a degree of mineralization in the range 0.25–0.43 [19].

Figs 6 and 7 and 8 show that for certain range of mineral inclinations, the fitting function is in agreement with the genetic algorithm results [14]. Whereas in Table 2 are reported the values of the standard deviation of the Gaussian PDF that allow the matching of it.

This interval of inclination could be due to the presence of the proximal collagen fibrils that causes a constriction of the mineral orientation [4, 7, 23, 44]. Overall, an increase of the degree of mineralization corresponds to a slightly wider range of apatite inclination with respect to the trabecula axes.

The diffusion coefficient D_L_ in the longitudinal direction of the trabecula matches the results of diffusivity from [14] for inclination of the apatite crystals in the LW plane characterized by a Gaussian PDF with mean 0 degrees and standard deviation close to 10 degrees (Fig 6A and 6B).

The diffusion coefficient decreases when the apatite minerals present configurations with a higher range of inclination. This situation is explained by an increased value of the tortuosity within inclined apatite platelets. In Fig 5A an example of a tortuous pathway, in both aligned and inclined apatite platelets, for a diffusion gradient parallel to the longitudinal axis in the LW plane is illustrated.

The Gaussian PDF of the apatite arrangement that allow an agreement with the genetic algorithm results [14] varies with the degree of mineralization.

Both cases of mineralization find agreement with Landis et al. [23] and Reznikov et al. [45] with regard to the range of mineral inclinations with respect to the longitudinal axis of trabecula. For a low degree of mineralization (i.e., V_f_A_ = 0.25), the platelets show a slightly major alignment with the longitudinal axis of trabecula. The preferential orientation achieved for low mineralized tissue could be explained by the deposition of mineral during the first phase of mineralization within the gap zones of the fibrils. Assuming that, in this case the collagen does not represent a limit for the mineral arrangements and the crystals lie along the lines of mechanical stress.

The diffusivity D_L_, expressed in function of the apatite inclination in the LT plane is represented in S1 Fig. It can be observed a slightly minor dependence of the diffusivity from the mineral inclination than in the previous case.

The diffusion coefficient D_W_ along the width direction of the trabecula is represented in function of the standard deviation σ_LW_ of the platelet inclination in the LW plane (Fig 7A and 7B).

The diffusivity enhances its value for arrangements of mineral characterized by higher standard deviations. This is justified by a smaller value of tortuosity in presence of inclined apatite platelets. In S4 Fig, a comparison pattern between a longer tortuous flowpaths in aligned mineral matrix than in an inclined apatite configuration is shown for a diffusion gradient in the width direction of the LW plane.

Depending on the degree of mineralization, the range of inclinations that allows the matching between the present model and the previous data [14], increases with the increasing mineral content. Furthermore, it can be noticed a major dependence of the diffusion coefficient on the range of apatite inclination in the case of a lower mineralization, i.e. V_f_A_ = 0.25.

The diffusion coefficient D_T_ parallel to the thickness direction of the trabecula is represented as a function of the standard deviation σ_LT_ of the inclination Gaussian PDF in the LT plane (Fig 8A and 8B). For both mineralization values, V_f_A_ = 0.25 and V_f_A_ = 0.43, a variation of the inclination range in this plane affects considerably the diffusion coefficient D_T_. For an increment of the apatite inclination interval, higher values of the diffusivity are achieved. This trend can be explained by the less tortuous path the water molecule should perform between two fixed extremes in presence of inclined platelets. In Fig 5B the previous situation is illustrated.

In the WT plane (S2 Fig and S3 Fig), the variation of θ_WT_ from the aligned orientation has a restrained influence on the diffusion coefficient for both directions and for all the mineralization degrees considered. The diffusion coefficients D_W_ and D_T_, present, also in this plane, an increasing trend for wider range of apatite inclination. In fact, the tortuosity in the WT plane decreases when it is considered within a mineral configuration highly inclined with respect to the axes of the trabecula coordinate system (Fig 5C).

Although in the present work the 3D geometric model excludes the effect chemical bonds between water and apatite platelets and also local changes within the native collagen, the results are in good agreement with [46] achieved by means of molecular dynamics simulations.

The differences could be due to a) the analysis that do not consider the complex chemical interactions or the subtle changes of the order of 1.0 Å [47] due to dehydratation; b) the different water diffusion coefficient in free water.

In summary, from a mass transport point of view, the diffusivity results obtained from the Monte Carlo method are according to the diffusion coefficients obtained by means of the genetic algorithm [14] and the molecular dynamics simulations [46]. It is worth pointing out that transport phenomena studies provide a deeper understanding of the physical and biological properties of bone at the nanoscale.

Understanding bone’s nanostructure may allow new insight of mimicking mechanical and ultra structural property of mineralized collagen fibril. The results presented here will be helpful in the study of bone pathologies and the developing of biomimetic strategies for tissue engineering.

## Supporting information

S1 Fig Diffusion coefficients D_L_ versus the standard deviation (σ_θ_LT_) of the Gaussian PDF that characterizes the apatite platelets inclination in the LT plane. The color bands represent the Confidence Interval at 95 percent. We reported also two different degrees of mineralization (V_f_A_ = 0.25, and V_f_A_ = 0.43). The continuous black line represent the Diffusion coefficient computed by means of a genetic algorithm [14].
(TIF)

S2 Fig Diffusion coefficients D_W_ versus the standard deviation (σ_θ_WT_) of the Gaussian PDF that characterizes the apatite platelets inclination in the WT plane. The color bands represent the Confidence Interval at 95 percent. We reported also two different degrees of mineralization (V_f_A_ = 0.25, and V_f_A_ = 0.43). The continuous black line represent the Diffusion coefficient computed by means of a genetic algorithm [14].
(TIF)

S3 Fig Diffusion coefficients D_T_ versus the standard deviation (σ_θ_WT_) of the Gaussian PDF that characterizes the apatite platelets inclination in the WT plane. The color bands represent the Confidence Interval at 95 percent. We reported also two different degrees of mineralization (V_f_A_ = 0.25, and V_f_A_ = 0.43). The continuous black line represent the Diffusion coefficient computed by means of a genetic algorithm [14].
(TIF)

S4 Fig Schematic picture of the streamlines involved in the calculation of the tortuosity factor in the LW plane, for a diffusion gradient in the width W direction of the trabecula coordinate system. Two examples of aligned (θ = 0 degrees) and inclined (θ = 20 degrees) apatite platelets configurations are shown. The blue dashed lines represent the path of water molecule within the aligned apatite matrix whilst the green dashed line indicates the pathway in the inclined mineral matrix. The latter is also reported in the aligned configuration of the apatite in order to facilitate the comparison between the two path lengths. The red continuous lines indicate the Euclidean distance between the path extremes.
(TIF)
