# Supplementary figures and images for: 3D diffusion model within the collagen apatite porosity: An insight to the nanostructure of human trabecular bone
Source: PLoS One. 2017 Dec 8;12(12):e0189041. doi: 10.1371/journal.pone.0189041 (PMC5722326; doi:10.1371/journal.pone.0189041)

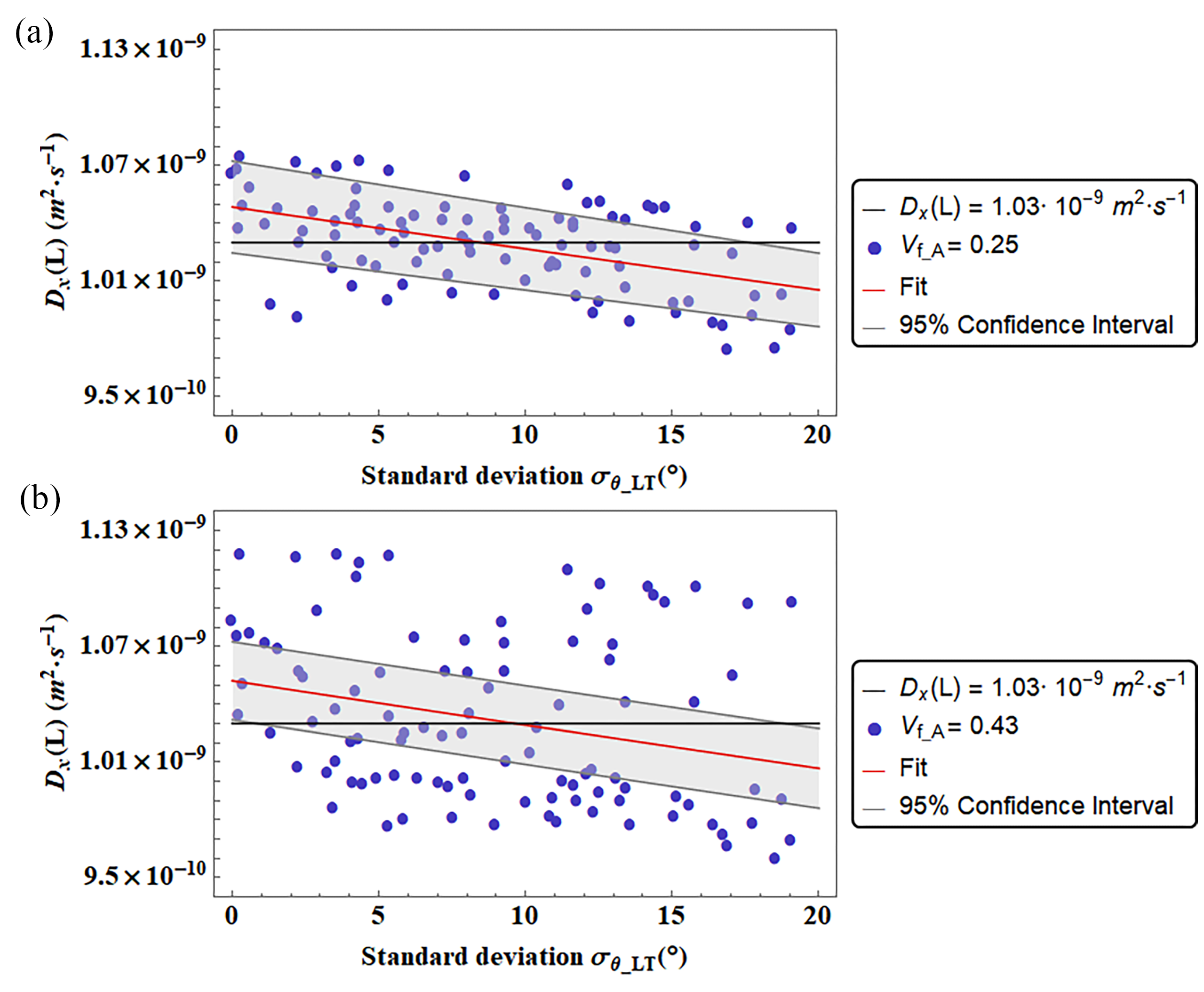

Supplement: S1 Fig — The color bands represent the Confidence Interval at 95 percent. We reported also two different degrees of mineralization (Vf_A = 0.25, and Vf_A = 0.43). The continuous black line represent the Diffusion coefficient computed by means of a genetic algorithm [14]. (TIF) [file pone.0189041.s001.tif]

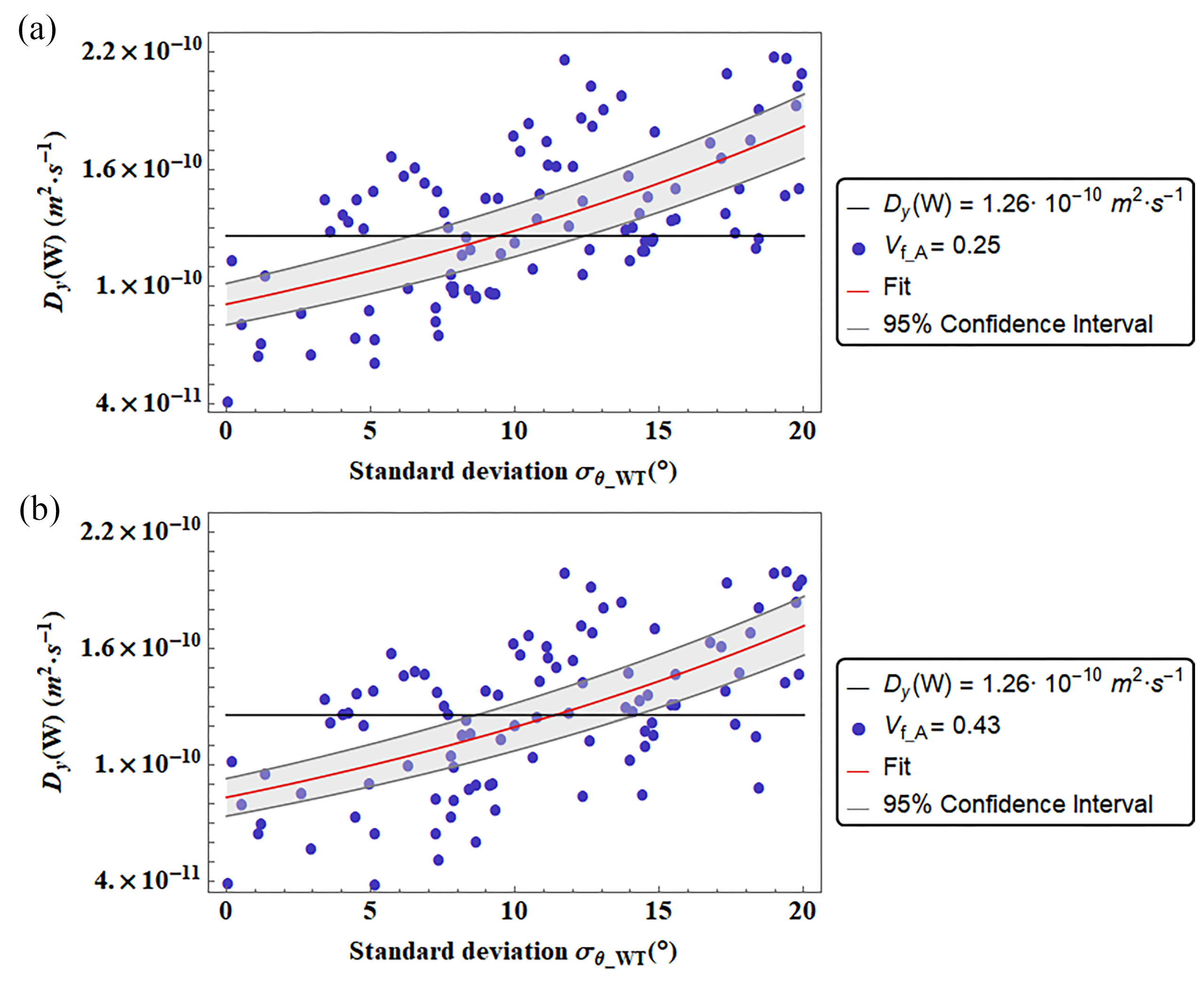

Supplement: S2 Fig — The color bands represent the Confidence Interval at 95 percent. We reported also two different degrees of mineralization (Vf_A = 0.25, and Vf_A = 0.43). The continuous black line represent the Diffusion coefficient computed by means of a genetic algorithm [14]. (TIF) [file pone.0189041.s002.tif]

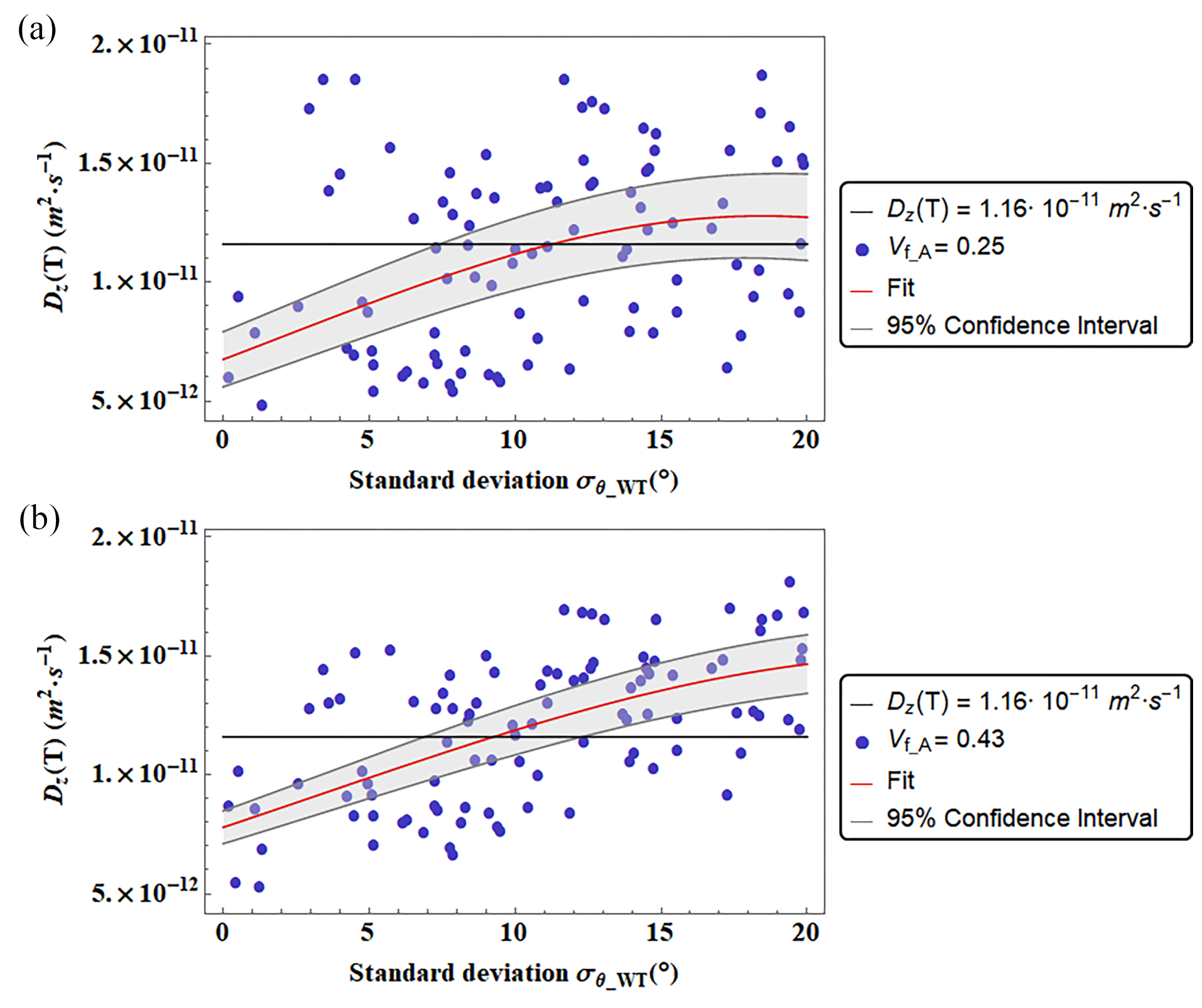

Supplement: S3 Fig — The color bands represent the Confidence Interval at 95 percent. We reported also two different degrees of mineralization (Vf_A = 0.25, and Vf_A = 0.43). The continuous black line represent the Diffusion coefficient computed by means of a genetic algorithm [14]. (TIF) [file pone.0189041.s003.tif]

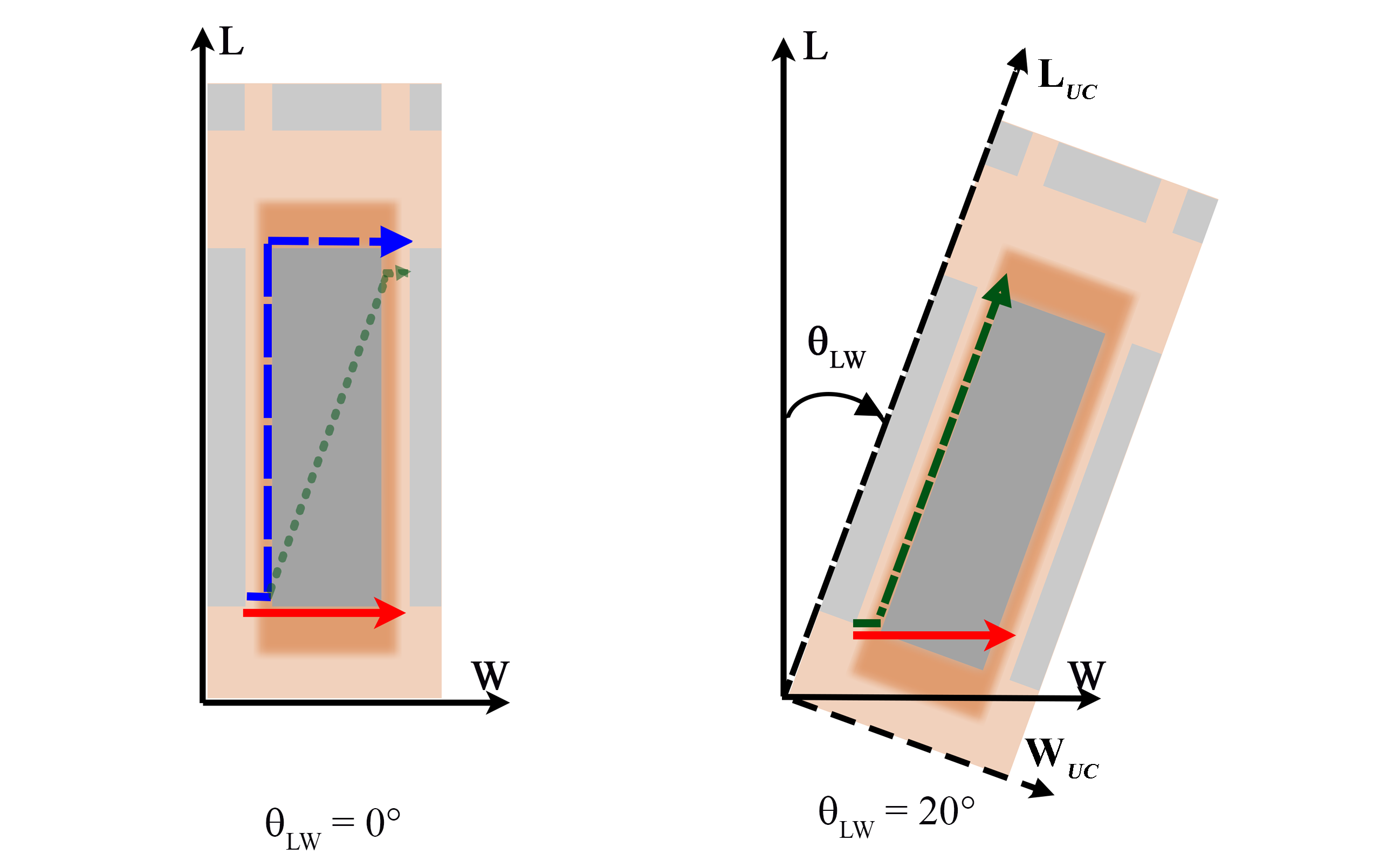

Supplement: S4 Fig — Two examples of aligned (θ = 0 degrees) and inclined (θ = 20 degrees) apatite platelets configurations are shown. The blue dashed lines represent the path of water molecule within the aligned apatite matrix whilst the green dashed line indicates the pathway in the inclined mineral matrix. The latter is also reported in the aligned configuration of the apatite in order to facilitate the comparison between the two path lengths. The red continuous lines indicate the Euclidean distance between the path extremes. (TIF) [file pone.0189041.s004.tif]
